# Glycemic variability determined with a continuous glucose monitoring system can predict prognosis after acute coronary syndrome

**DOI:** 10.1186/s12933-018-0761-5

**Published:** 2018-08-18

**Authors:** Hironori Takahashi, Noriaki Iwahashi, Jin Kirigaya, Shunsuke Kataoka, Yugo Minamimoto, Masaomi Gohbara, Takeru Abe, Kozo Okada, Yasushi Matsuzawa, Masaaki Konishi, Nobuhiko Maejima, Kiyoshi Hibi, Masami Kosuge, Toshiaki Ebina, Kouichi Tamura, Kazuo Kimura

**Affiliations:** 1 Division of Cardiology, Yokohama City University Medical Center, 4-57 Urafune-cho, Minami-ku, Yokohama, 232-0024 Japan; 2 Advanced Critical Care and Emergency Center, Yokohama City University Medical Center, 4-57 Urafune-cho, Minami-ku, Yokohama, Japan; 3 Department of Medical Science and Cardiorenal Medicine, Yokohama City University Graduate School of Medicine, 3-9 Fukuura, Kanazawa-ku, Yokohama, Japan

## Abstract

**Background:**

Impaired glucose metabolism is an established risk factor for coronary artery disease. Previous studies revealed that glycemic variability (GV) is also important for glucose metabolism in patients with acute coronary syndrome (ACS). We explored the association between GV and prognosis in patients with ACS.

**Methods:**

A total of 417 patients with ACS who received reperfusion wore a continuous glucose monitoring system (CGMS) in a stable phase after admission and were monitored for at least 24 consecutive h. The mean amplitude of glycemic excursion (MAGE) was calculated as a marker of GV. We divided into two groups based on the highest tertile levels of MAGE (MAGE = 52 mg/dl). The groups were followed up for a median of 39 months [IQR 24–50 months]. The primary endpoint was the incidence of major adverse cardiovascular and cerebrovascular events (MACCE).

**Result:**

During follow-up, 66 patients experienced MACCE (5 patients had cardiovascular death, 14 had recurrence of ACS, 27 had angina requiring revascularization, 8 had acute decompensated heart failure, and 16 had a stroke). MACCE was more frequently observed in the high MAGE group (23.5% vs. 11.6%, *p* = 0.002). In multivariate analysis, high MAGE was an independent predictive factor of poor prognosis for MACCE (odds ratio, 1.84; 95% confidence interval, 1.01–3.36; *p *= 0.045).

**Conclusion:**

Glycemic variability determined with a CGMS is a predictor of prognosis in patients with ACS without severe DM.

*Trial registration* UMIN 000010620. Registered April 1st 2012

## Background

Impaired glucose metabolism is an established risk factor for coronary artery disease [1]. Patients with diabetes mellitus (DM) have increased mortality rates and a two to three times higher risk of cardiovascular disease as compared with patients with no history of DM [2]. Previous studies showed that a higher glucose level on admission [3], hypoglycemia during hospitalization [4], and sustained hyperglycemia, as determined by glycosylated hemoglobin A1c (HbA1c) [5], were markers of poor prognosis for acute myocardial infarction (AMI). As reported earlier, glycemic variability (GV) has specific clinical implications, as well as a different meaning compared with that of classical markers [6, 7]. A continuous glucose monitoring system (CGMS) is an emerging technology that can continuously measure glucose levels, thereby enabling evaluation of GV. We previously reported that GV measured with a CGMS in the stable phase of ST-elevation myocardial infarction (STEMI) predicted left ventricular remodeling, as determined by cardiac magnetic resonance imaging (CMRI) [8]. It has been reported that GV was predictive of mortality in elderly patients with AMI [9]. Furthermore, we also reported that GV predicted rapid progression of coronary plaque in patients with acute coronary syndrome (ACS) [10]. Moreover, we had already reported that GV had a significant association with the vulnerability of plaque [8, 11]. However, the impact on the prognosis of GV in patients with ACS still remains unclear. Therefore, we explored the effect of GV on prognosis in patients with ACS during long-term follow-up.

## Methods

### Study population

**Fig. 1:**
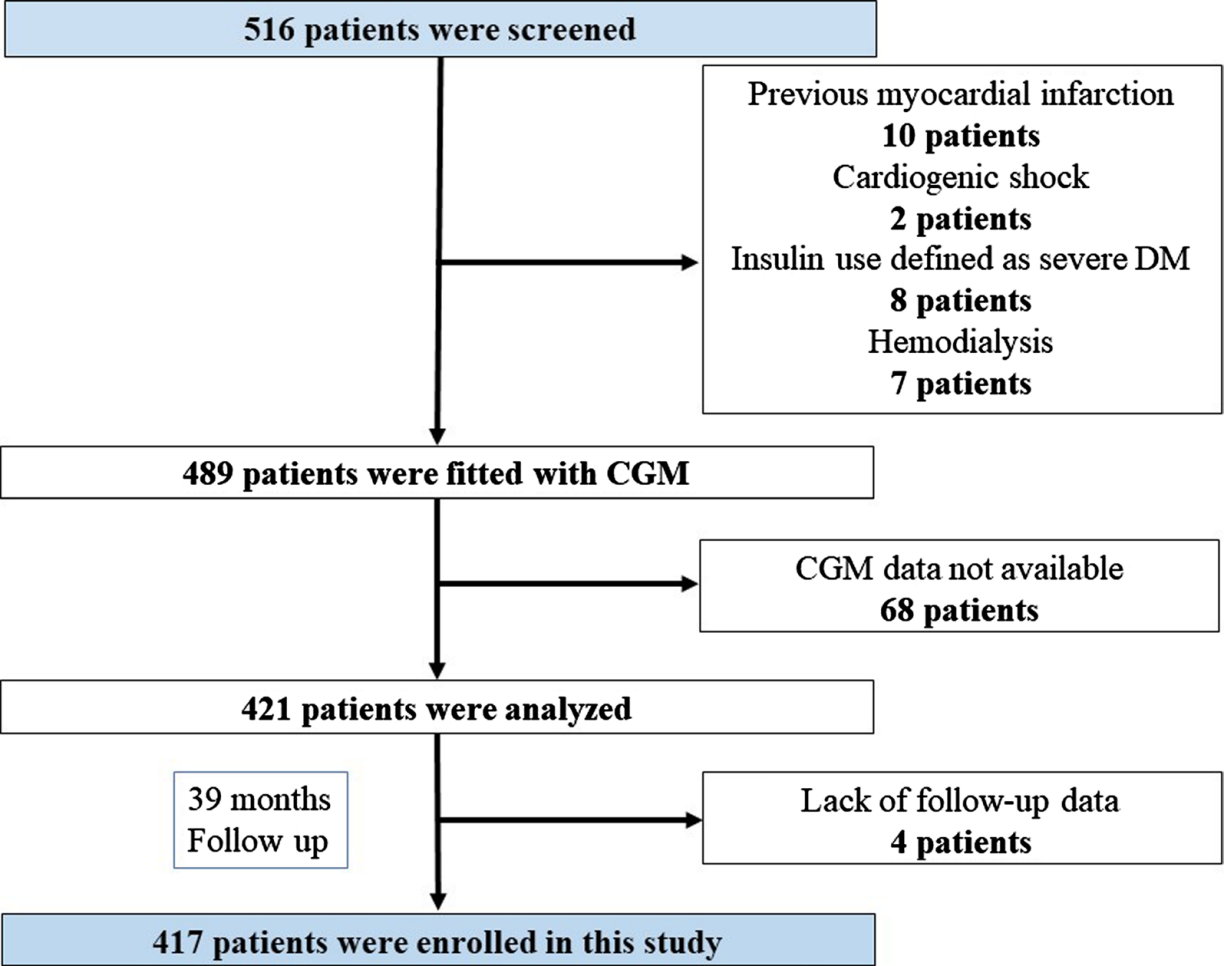
Flow chart of enrolment in this study of GV determined by continuous glucose monitoring for prediction of prognosis following ACS

We studied 516 patients with ACS who underwent percutaneous coronary intervention (PCI) in Yokohama City University Medical Center between April 2012 and November 2016. ACS was defined as ST-segment elevation acute coronary syndrome (STE-ACS) and non-ST-segment elevation acute coronary syndrome (NSTE-ACS) [12]. Patients fulfilling any of the following criteria were excluded: previous myocardial infarction (n = 10), cardiogenic shock (n = 2), insulin use defined as severe DM (n = 8), hemodialysis (n = 7), CGMS data not available (n = 68), or lack of follow-up data (n = 4). A total of 417 patients with first ACS were enrolled (Fig. 1). Admission hyperglycemia was defined as admission plasma glucose level > 180 mg/dl [13]. Hypertension was defined as systolic blood pressure > 130 mmHg or diastolic blood pressure > 80 mmHg [14] or treatment with oral antihypertensive drugs. Hypercholesterolemia was defined as low-density lipoprotein cholesterol ≥ 140 mg/dl [15] or treatment with oral antihypercholesterolemic drugs. All patients underwent calculation of the global registry of coronary events (GRACE) score, and a high GRACE score was defined as > 140 based on previous reports [12]. The study protocol was approved by the Yokohama City University Medical Center Institutional Review Board, and all patients gave written informed consent. (UMIN-CTR ID: UMIN000010620).

### Blood sampling

Peripheral blood samples, including blood glucose, creatinine phosphokinase (CPK), and creatine kinase MB (CK-MB) levels, were collected after admission and at 3-h intervals during the first 24 h. Brain natriuretic peptide (BNP) and high-sensitivity C-reactive protein (hs-CRP) were evaluated on admission, daily until discharge, and 1 month after the onset of ACS in the stable phase. We divided all patients into two groups according to hs-CRP greater or less than 0.1355 mg/dl, as an indicator of predicted rapid progression of coronary artery disease in a prior study [10]. Patients with any conditions (cancer and inflammatory disease) known to modify hs-CRP levels were excluded from the assessment of hs-CRP. Biochemical markers were evaluated at the time of admission, and stable phase values were recorded.

### CGMS protocol

All patients were fitted with a CGMS (i Pro2, Medtronic, Minneapolis, MN, USA) and were monitored for at least 24 consecutive hours during a stable state when they could take three regular meals. The CGMS sensor was inserted into subcutaneous abdominal fat tissue. During CGMS, blood glucose levels were checked at least four times per day, using a self-monitoring blood glucose device (Medisafe Mini; Terumo, Japan) to calibrate the CGMS data. The data obtained by the CGMS were recorded and analyzed off-line.

**Fig. 2:**
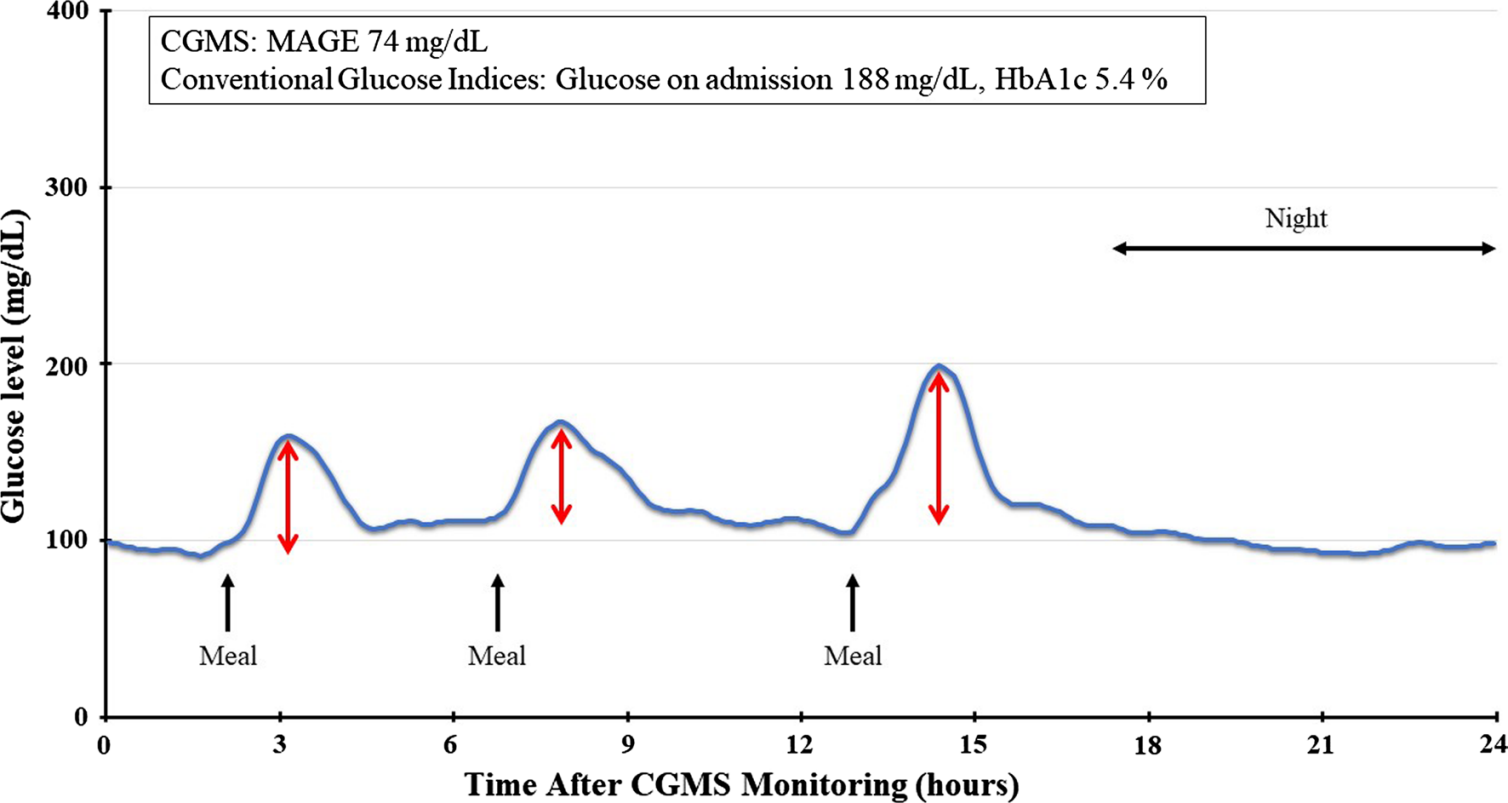
Representative case of use of the CGMS. The patient was an 86-year-old man who was diagnosed with anterior STE-ACS. He had IGT on a 75-g oral glucose tolerance test during hospitalization. His conventional glucose indicators showed admission hyperglycemia and HbA1c 5.4%. The CGMS can visualize GV. The MAGE is calculated by measuring the arithmetic mean of the difference between consecutive peaks and nadirs (red arrows) if the difference is > 1 SD of the mean glucose. The CGMS revealed that the MAGE was 74 mg/dl. He died after being hospitalized for heart failure 9 months later

The results were interpreted by two experienced observers. The average glucose level (Ave) and standard deviation (SD) and the coefficient of variation (CV) were calculated, in addition to the mean amplitude of glycemic excursion (MAGE). The MAGE was determined by calculating the arithmetic mean of the difference between consecutive peaks and nadir if the difference was > 1 SD of the mean glucose level [16]. Figure 2 shows a representative case of CGMS monitoring. The conventional glucose indicators showed admission hyperglycemia and HbA1c 5.4%, i.e. within normal range. CGMS monitoring revealed MAGE of 74 mg/dl. We divided all 417 patients into two groups according to the MAGE levels. Patients belonging to the highest tertile of MAGE were categorized into the high MAGE group and the other two-thirds into the low MAGE group. The optimal cut-off point of MAGE was also consistent with the value determined by the Youden index, i.e. J = max (sensitivity + specificity − 1) [17].

### 75-g oral glucose tolerance test protocol

All patients who had not been given a diagnosis of DM underwent a standard 75-g oral glucose tolerance test between the 4th hospital day and discharge, after their condition had been stabilized. After an overnight fast, venous blood samples for the measurement of plasma glucose were taken at baseline and 30 min, 60 min, and 120 min after an oral glucose load. DM, impaired glucose tolerance (IGT), and normal glucose tolerance (NGT) were classified according to the criteria of the American Diabetes Association.

### Long-term follow-up and definitions of major adverse cardiovascular and cerebrovascular events (MACCE)

Patients were followed up for a mean period of 39 months [IQR 24–50 months]. During follow-up, this study used a composite MACCE defined as the occurrence of one of the following events: cardiac death, recurrence of ACS, angina requiring revascularization, acute decompensated heart failure (ADHF) requiring hospitalization, and stroke. Angina requiring revascularization was defined as having an indication for coronary artery revascularization [18]. All events were followed up by a hospital visit or telephone interview with an experienced cardiovascular physician blinded to clinical details and outcomes.

### Statistical analysis

Continuous data were expressed as median (interquartile range), and categorical data were reported as frequencies and percentages. First, we used univariate analysis to identify associations between MAGE groups and all variables as follows: all baseline characteristics (age, sex, body mass index, STE-ACS, Killip class > 1, GRACE score > 140, infarct-related artery, multivessel disease, hypertension, hypercholesterolemia, systolic blood pressure on admission, heart rate, DM, IGT, NGT, medication on discharge) and laboratory data [creatinine, estimated glomerular filtration rate, peak level of CPK, BNP during stable phase, hs-CRP during stable phase > 0.1355 mg/dl, low-density lipoprotein cholesterol, high-density lipoprotein (HDL) cholesterol, triglycerides, glucose on admission, glucose on admission > 180 mg/dl, HbA1c], and CGM findings (MAGE, Ave, SD, CV). Student’s t-test was used to compare differences in continuous variables among groups. For categorical variables, Fisher’s exact test or the Chi squared test was used, as appropriate. Second, we utilized univariate logistic regression models for the prediction of MACCE with all variables. Third, to control for effects of confounding factors, we adopted three, stepwise, multiple logistic regression models for the prediction of MACCE with all independent variables, with *p* < 0.05 in the univariate analysis. Model 1 included multivessel disease, BNP during stable phase, hs-CRP during stable phase > 0.1355 mg/dl, and HDL cholesterol; Model 2 included glucose on admission > 180 mg/dl, HbA1c, and high MAGE; Model 3 included all variables included in Model 1 and 2. Lastly, we used the area under the curve (AUC), and 95% confidence interval (CI), for each model, and tested increments of AUC from Model 1 to 2 or 3 with the Delong method [19]. In addition, we performed sensitivity analysis to validate main findings, using subgroups of the DM and IGT patients. For each group, we utilized univariate logistic regression models for the prediction of MACCE with all independent variables, and adopted multiple logistic regression models for the prediction of MACCE with independent variables which were obtained from the univariate analysis with *p* < 0.05. A *p*-value < 0.05 in a two-tailed test was considered statically significant. All statistical analyses were performed using JMP, version 12.0.0 (SAS Institute Inc., Cary, NC, USA) and MedCalc Statistical Software version 16.4.3 (MedCalc Software bvba, Ostend, Belgium; https://www.medcalc.org; 2016).

## Results

### Baseline characteristics

The characteristics of all patients are stratified in Tables 1, 2. The mean age was 66 years (interquartile range [IQR 56–74 years]), and 83% of the patients were male. In the study, 61% had a medical history of hypertension, 38% had hypercholesterolemia, 140 (34%) had DM, 185 (44%) had IGT, and 92 (22%) had NGT. Median HbA1c level was 5.9% in all patients; however, when we limited our study to DM patients, median HbA1c was 6.7% [IQR 6.1–7.7%]. Except for glucose metabolism, significant differences between two groups were observed in body mass index, Killip class > 1, multivessel disease, and BNP during stable phase.

**Table 1:** Baseline clinical characteristics

Variables	All patients	Low MAGE	High MAGE	*p*-value
	(n = 417)	(n = 268)	(n = 149)	
Age, years	66 (56–74)	66 (56–74)	68 (58–75)	0.275
Male, n (%)	348 (83)	219 (82)	129 (85)	0.201
Body mass index, kg/m^2^	26.8 (22.1–29.5)	24.5 (22.4–27.3)	23.9 (21.8–26.1)	0.010
STE-ACS, n (%)	292 (70)	191 (71)	101 (68)	0.457
Killip class > 1, n (%)	74 (18)	40 (15)	34 (23)	0.042
Infarct-related artery, n (%)				
Left anterior descending coronary artery, n (%)	229 (55)	150 (56)	79 (53)	0.562
Left circumflex coronary artery, n (%)	45 (11)	29 (11)	16 (11)	0.979
Right coronary artery, n (%)	143 (34)	89 (33)	54 (36)	0.532
Multivessel disease, n (%)	176 (42)	99 (37)	77 (52)	0.004
GRACE score > 140, n (%)	212 (51)	125 (47)	87 (58)	0.022
Hypertension, n (%)	255 (61)	155 (58)	100 (67)	0.063
Hypercholesteremia, n (%)	160 (38)	109 (41)	51 (34)	0.195
Systolic blood pressure on admission, mmHg	150 (125–170)	149 (125–167)	153 (124–177)	0.806
Heart rate on admission, bpm	76 (64–88)	76 (64–88)	76 (65–90)	0.575
Laboratory data				
Cre on admission, mg/dl	0.84 (0.72–1.00)	0.84 (0.73–0.98)	0.83 (0.71–1.02)	0.720
eGFR on admission, %	67.9 (56.3–81.1)	67.3 (55.9–80.0)	70.4 (56.3–83.1)	0.091
Peak level of CPK, IU/l	1129 (250–2864)	1130 (243–2870)	1129 (265–2838)	0.696
BNP during stable phase, pg/ml	89.2 (35.5–192.7)	80.4 (35.4–171.4)	101.3 (37.7–209.1)	0.007
hs-CRP during stable phase > 0.1355 mg/dl, n (%)	185 (44)	115 (43)	70 (47)	0.393
Lipid profile on admission				
LDL cholesterol, mg/dl	128 (105–152)	131 (108–155)	124 (102–148)	0.114
HDL cholesterol, mg/dl	44 (37–52)	43 (37–51)	45 (37–52.5)	0.131
Triglycerides, mg/dl	118 (75–185)	124 (78–197)	113 (70–162)	0.125
Medication on discharge, n (%)				
ACE-I or ARB	334 (80)	210 (78)	124 (83)	0.233
β-blocker	275 (66)	175 (65)	100 (67)	0.708
Statin	400 (96)	258 (96)	142 (95)	0.632

*STE-ACS* ST-segment elevation acute coronary syndrome, *GRACE score* grobal registry of coronary event, *Cre* creatinine, *eGFR* estimated glomerular filtration rate, *CPK* creatine phosphokinase, *BNP* B-type natriuretic peptide, *hs-CRP* high sensitivity C-reactive protein, *LDLC* low-density lipoprotein cholesterol, *HDLC* high-density lipoprotein cholesterol, *ACE-I* angiotensin-converting enzyme-inhibitors, *ARB* angiotensin II receptor blockers

**Table 2:** Baseline characteristics of glycemic metabolism

Variables	All patients	Low MAGE	High MAGE	*p*-Value
	(n = 417)	(n = 268)	(n = 149)	
75 g OGTT findings				
Diabetes mellitus, n (%)	140 (34)	55 (21)	85 (57)	< 0.001
Impaired glucose tolerance, n (%)	185 (44)	135 (50)	50 (34)	< 0.001
Normal glucose tolerance, n (%)	92 (22)	78 (29)	14 (9)	< 0.001
Glucose on admission, mg/dl	142 (118–182)	133 (114–165)	163 (130–217)	< 0.001
Glucose on admission > 180 mg/dl	105 (25)	49 (18)	56 (38)	< 0.001
Hemoglobin A1c level, %	5.9 (5.5–6.4)	5.8 (5.5–6.1)	6.3 (5.7–7.3)	< 0.001
CGM findings				
MAGE, mg/dl	40.6 (27.59.5)	31.8 (24.39.7)	65.0 (57.8–79)	< 0.001
Ave, mg/dl	120 (108–136)	115 (106–126)	134 (120–170)	< 0.001
SD	17.0 (12.1–25.1)	13.5 (10.5–17.2)	28 (22.4–35.9)	< 0.001
CV	14.0 (10.2–18.7)	11.3 (9.1–14.2)	19.7 (16.4–23.6)	< 0.001

*OGTT* oral glucose tolerance test, *CGM* continuous glucose monitoring, *MAGE* mean amplitude of glycemic excursions, *Ave* average, *SD* standard deviation, *CV* coefficient of variation

### Incidence of MACCE

**Fig. 3:**
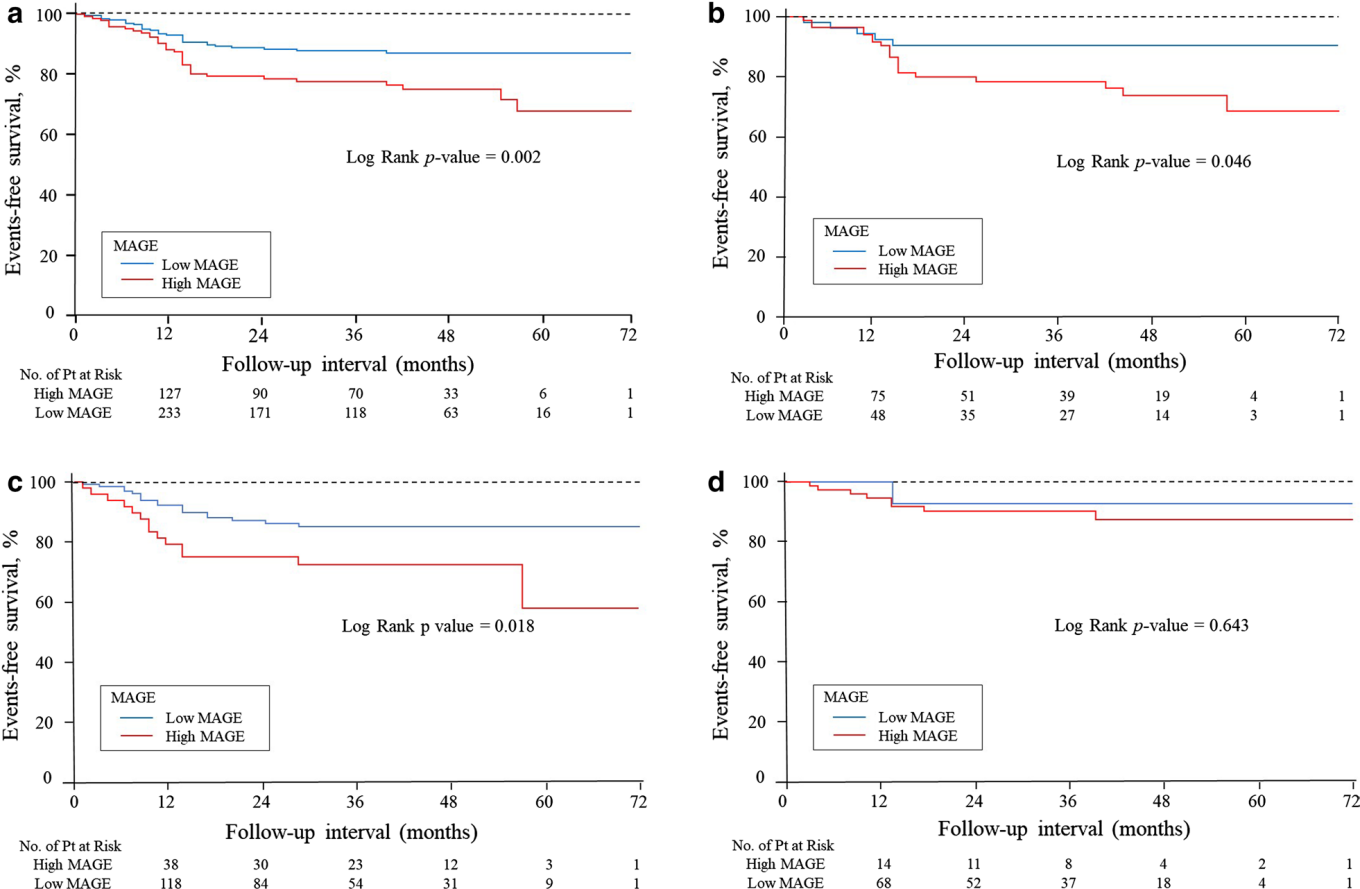
Kaplan-Meier survival for patient group by MAGE. The cut-off value defining MAGE was 52 mg/dl. The red line indicates the high MAGE group. The high MAGE group had a significantly lower event-free survival rate (**a**). When we limited patients to those with DM (**b**) and IGT (**c**), high MAGE was a significant predictor. In patients with NGT, the high MAGE did not correlate with the prognosis (**d**)

During follow-up, 66 patients (16%) experienced MACCE: 5 (1.2%) had cardiovascular death, 14 (3.4%) had recurrence of ACS, 27 (6.5%) had angina requiring revascularization, 8 (1.9%) had heart failure, and 16 (3.8%) had stroke. Kaplan–Meier curves for patients by MAGE are shown in Fig. 3. The high MAGE group had a significantly lower event-free survival rate (a) (*p *= 0.002). Even if limited to patients with DM (n = 140) (b) or IGT (n = 185) (c), the results were similar. In patients with NGT (n = 92), the high MAGE group did not correlate with the prognosis (d).

### Prediction of MACCE

Table 3 shows associations between the prediction of MACCE and all variables by univariate analysis. Significant associations were found between the prediction of MACCE and high MAGE, multivessel disease, BNP level during stable phase, hs-CRP > 0.1355 mg/dl, HDL cholesterol level, glucose on admission > 180 mg/dl, and HbA1c level. Table 4 shows multivariate analysis for the prediction of MACCE. In Model 1, multivessel disease and hs-CRP > 0.1355 mg/dl were significant predictors of MACCE. MAGE was found to be an independent predictor of MACCE in Model 2 (odds ratio [OR], 2.030, 95% CI, 1.159–3.563; *p* = 0.014) and Model 3 (OR, 1.844; 95% CI, 1.013–3.356; *p* = 0.045). Among these parameters, MAGE was an independent predictor of prognosis in patients with ACS. We estimated the AUC in Model 1 (AUC 0.68; 95% CI, 0.629–0.722), Model 2 (AUC, 0.63; 95% CI, 0.582–0.678), and Model 3 (AUC, 0.72; 95% CI, 0.676–0.765). We found that an AUC increment of 0.05 from Model 1 to Model 2 was not significant (95% CI, − 0.052–0.144, *p *= 0.358), and that an increment of 0.05 in Model 3 showed a tendency toward significance (95% CI, − 0.000–0.091; *p *= 0.055) (Table 5). Table 6 shows the results of the sensitivity analysis. Regarding MACCE, MAGE was the most significant predictor among patients with DM (OR, 3.238; 95% CI, 1.041–12.383; p = 0.042). There were three significant variables in patients with IGT (Multivessel disease, hs-CRP, High MAGE). MAGE had the tendency to act as a prognosticator among patients with IGT (OR, 2.080, 95% CI, 0.861–4.957; p = 0.102). Please see Table 6 for further information.

**Table 3:** Univariate logistic regression analysis for the prediction of MACCE in ACS patients

Variables	OR	95% CI	*p*-value
Age, per 1 year	1.007	0.985–1.031	0.511
Male	0.990	0.489–2.005	0.978
Body mass index, per 1 kg/m^2^	0.943	0.872–1.015	1.060
STE-ACS	0.831	0.474–1.456	0.559
Killip class > 1	1.028	0.520–2.037	0.935
Culprit LAD	0.982	0.579–1.666	0.947
Culprit LCx	0.488	0.169–1.411	0.177
Cilprit RCA	1.300	0.757–2.234	0.341
Multivessel disease	0.411	0.240–0.704	0.001
GRACE score > 140	1.283	0.756–2.178	0.355
Hypertension	0.838	0.492–1.429	0.516
Hypercholesterolemia	1.053	0.614–1.804	0.852
Systolic blood pressure on admission, per 1 mmHg	0.995	0.988–1.002	0.214
Heart rate on admission, per 1 bpm	1.003	0.990–1.017	0.638
Cre on admission, per 1 mg/dl	1.598	0.909–2.914	0.098
eGFR on admission, per 1%	0.996	0.982–1.008	0.522
Peak level of CPK, per 1 IU/l	1.000	0.999–1.000	0.534
BNP during stable phase, per 1 pg/ml	1.001	1.000–1.003	0.013
hs-CRP during stable phase > 0.1355 mg/dl	2.278	1.325–3.915	0.002
LDL cholesterol, per 1 mg/dl	1.000	0.993–1.007	0.994
HDL cholesterol, per 1 mg/dl	0.972	0.946–0.996	0.030
Triglycerides, per 1 mg/dl	0.999	0.997–1.001	0.571
ACE-I or ARB use at discharge	0.737	0.396–1.374	0.336
β-blocker use at discharge	1.127	0.642–1.979	0.676
Statin use at discharge	0.596	0.188–1.888	0.374
Diabetes mellitus	1.251	0.726–2.158	0.420
Impaired glucose tolerance	1.218	0.719–2.063	0.463
Normal glucose tolerance	0.510	0.242–1.074	0.072
Glucose on admission > 180 mg/dl	2.238	1.286–3.893	0.004
Hemoglobin A1c level, %	1.267	1.030–1.546	0.021
High MAGE	2.347	1.378–3.998	0.001
Ave, mg/dl	1.001	0.998–1.013	0.155
SD	1.016	0.994–1.038	0.141
CV	1.020	0.982–1.058	0.293

*LAD* left anterior descending coronary artery, *LCx* left circumflex coronary artery, *RCA*, right coronary artery, *OR* odds ratio, *95% CI* 95% confidence interval. Other abbreviations as in Tables 1, 2

**Table 4:** Multiple logistic regression analysis for the prediction of MACCE in ACS patients

Variables	Multivariate (Model 1)			Multivariate (Model 2)			Multivariate (Model 3)		
	OR	95% CI	*p*-value	OR	95% CI	*p*-value	OR	95% CI	*p*-value
Multivessel disease	2.251	1.294–3.968	0.004		–		2.075	1.176–3.703	0.012
BNP during stable phase, per 1 pg/ml	1.001	0.999–1.003	0.104		–		1.001	0.999–1.002	0.232
hs-CRP during stable phase > 0.1355 mg/dl	2.026	1.166–3.575	0.012		–		2.103	1.196–3.761	0.010
HDLC, per 1 mg/dl	0.974	0.012–0.944	0.052		–		0.976	0.948–1.002	0.077
Glucose on admission > 180 mg/dl		–		1.798	0.920–3.439	0.085	1.723	0.869–3.349	0.118
HbA1c, per 1%		–		1.045	0.807–1.334	0.728	1.050	0.800–1.361	0.714
High MAGE		–		2.030	1.159–3.563	0.014	1.844	1.013–3.356	0.045

*Model 1* multivessel disease, BNP during stable phase, hs-CRP during stable phase > 0.1355 mg/dl and HDLC, *Model 2* Glucose on admission > 180 mg/dl, HbA1c and High MAGE, *Model 3* all variables included in Model 1 and 2. Other abbreviations as in Tables 1, 2, 3

**Table 5:** Area under the curve predictive of MACCE in ACS patients

Variables	AUC	95% CI	Increment of AUC vs Model 1	95% CI	*p*-value
Model 1	0.68	0.629–0.722	–	–	–
Model 2	0.63	0.582–0.678	0.05	− 0.052–0.144	0.358
Model 3	0.72	0.676–0.765	0.05	− 0.000–0.091	0.055

*AUC* area under the curve. Other abbreviations as in Tables 3, 4

**Table 6:** Sensitivity analysis of Multiple logistic regression analysis for the prediction of MACCE in ACS patients

Variables	Multivariate (Model 1)			Multivariate (Model 2)			Multivariate (Model 3)		
	OR	95% CI	*p*-value	OR	95% CI	*p*-value	OR	95% CI	*p*-value
Patients with DM									
Hypertension	0.435	0.157–1.145	0.092		–		0.438	0.105–6.510	0.105
Cre on admission, per 1 mg/dl	1.141	0.407–4.202	0.829		–		1.208	0.401–4.762	0.768
BNP during stable phase, per 1 pg/ml	1.002	0.999–1.005	0.053		–		1.002	0.999–1.005	0.072
HDLC, per 1 mg/dl	0.962	0.909–1.011	0.134		–		0.966	0.912–1.017	1.035
Glucose on admission > 180 mg/dl		–		2.415	0.861–7.232	0.094	2.038	0.660–6.666	0.217
HbA1c, per 1%		–		1.154	0.842–1.575	0.364	1.091	0.753–1.562	0.637
High MAGE		–		2.780	1.017–8.956	0.046	3.238	1.041–12.38	0.042
Patients with IGT									
Multivessel disease	4.586	2.028–10.843	< 0.001		–		4.036	1.747–9.694	0.001
hs-CRP during stable phase > 0.1355 mg/dl	3.766	1.641–9.226	0.002		–		3.857	1.664–9.576	0.001
High MAGE		–		2.528	1.145–5.581	0.024	2.080	0.861–4.957	0.102

Patients with DM: *Model 1* hypertension, Cre on admission, BNP during stable phase and HDLC, *Model 2* glucose on admission > 180 mg/dl, *HbA1c* high MAGE, *Model 3* all variables included in Model 1 and 2; Patients with IGT: *Model 1* multivessel disease and hs-CRP during stable phase > 0.1355 mg/dl, *Model 2* included only High MAGE, *Model 3* all variables included in Model 1 and 2. Other abbreviations as in Tables 1, 2, 3, 4

## Discussion

The results of this study showed that a high MAGE, as determined by a CGM, was an independent predictor of long-term poor prognosis in patients with ACS who had undergone PCI. A high MAGE was an independent predictor of MACCE based on forced inclusion multivariate analyses. This is the first study to reveal the role of GV, as evaluated by a CGM, on long-term prognosis during the current intervention era.

### The role of GV in coronary events

In this study, we demonstrated that GV was an important factor in the progression of coronary artery disease. Several mechanisms for our results, including oxidative stress, have been suggested to explain the role of GV in cardiovascular disease, and previous studies have shown that GV was a specific trigger for oxidative stress [20, 21]. It has been reported that oxidative stress promotes inflammation and endothelial dysfunction resulting in atherosclerosis [22]. Previous research has suggested that GV plays an important role in the development of complications related to impaired glucose metabolism. We previously reported several studies on the impact of GV on coronary plaque morphology and pathophysiology [23, 11] and noted that GV caused rapid plaque progression and adverse events [10]. Guideline for the management of acute-phase myocardial infarction recommend the blood glucose level should be kept above 90 mg/dl, but less than 200 mg/dl, and the goal is an HbA1c level of < 7.0% [24]. Based on our results, we emphasize that GV should be considered alongside these classic indexes.

### The role of GV in cardiovascular death and heart failure

Cardiovascular death and ADHF occurred in 13 patients, and we previously reported that GV predicts LV remodeling in patients with a first STEMI [8]. We consider that the result was mainly due to the characteristics of GV itself. GV has a more specific triggering effect on oxidative stress than sustained hyperglycemia [20], and it may be associated with these factors more strongly, thus affecting LV remodeling. In this study, the LV size and function measurements were performed using CMRI, which is believed to be the gold standard; therefore, our results included reliable data. We believe that our previous paper demonstrating that GV was an important factor in LV remodeling could explain this result [8]. Other studies concluded that GV also affected the variability in neointimal thickness after everolimus-eluting stent implantation in patients with coronary artery disease [25]. Su et al. reported that in-hospital MAGE may be an important predictor of mortality, and that MACE after AMI is a stronger predictor than HbA1c [9, 26]. However, in one of their papers they did not include the data regarding PCI, in another of theirs did not exclude the influence of the insulin use. Furthermore, we followed our patients for over 3 years, which was longer than the follow-up by Su et al. We believe that our study is the most reliable for the current PCI era, and conclude that our paper is the most useful regarding prognosis after ACS in terms of cardiac death and heart failure.

### The difference between GV and DM

Although both the HbA1c and GV may be associated with adverse prognoses, our study showed that an increased MAGE is more important than the HbA1c. An increased HbA1c represents long-term glucose regulation, whereas elevated GV suggests not only glucose dysregulation, but also stress and general poor health. We believe that the reason why GV was more important than the HbA1c was due mainly to the population in this study. There was a limited number of severe DM patients who underwent emergent PCI or who did not treat their DM before the target hospitalization. Therefore, the average HbA1c level was not high in this study (the median HbA1c level was 5.9%). In fact, a recent study did not include many diabetic patients [27]. Thus, we would like to emphasize that in patients without severe DM, GV seems to be a stronger predictor than HbA1c for prognosis in this population. We believe that this result is suitable for contemporary clinics.

### Clinical implications

Recent investigations have demonstrated that glucagon-like peptide-1 (GLP-1) analogue inhibits oxidative injuries in vascular endothelial cells [28]. Another study suggested that the α-glucosidase inhibitor attenuated GV, heart rate variability, and sympathetic activity in ACS patients with type 2 DM [29]. According to the current study’s results, we emphasize that there is a possibility of improving prognosis by reducing GV via the use of CGM. In the future, the real significance of GV by CGM in patients with ACS would be resolved by an intervention study.

### Study limitations

The present study did have some limitations. First, this was a small, prospective, observational trial conducted at a single center. Second, we excluded high-risk patients, including those with hemodialysis or clinical instability, such as cardiogenic shock. Third, we excluded patients treated with insulin. Although these limitations made our results more robust, those patients are at high risk; therefore, we would like to examine such patients in the next study.

## Conclusion

GV, as determined by a CGM, is a predictor of poor prognosis in patients with ACS without severe DM. Further studies are needed to clarify the clinical significance of GV in patients with ACS.

## Abbreviations

MAGE: mean amplitude of glycemic excursion
DM: diabetes mellitus
HbA1c: glycosylated hemoglobin As1c
AMI: acute myocardial infarction
GV: glycemic variability
CGMS: continuous glucose monitoring system
STEMI: ST-elevation myocardial infarction
CMRI: cardiac magnetic resonance imaging
ACS: acute coronary syndrome
PCI: percutaneous coronary intervention
CPK: creatinine phosphokinase
CK-MB: creatine kinase MB
BNP: brain natriuretic peptide
hs-CRP: high-sensitivity C-reactive protein
IGT: impaired glucose tolerance
NGT: normal glucose tolerance
ADHF: acute decompensated heart failure
MACCE: major adverse cardiac and cerebrovascular events
